# Factors Hindering Access and Utilization of Maternal Healthcare in Afghanistan Under the Taliban Regime: A Qualitative Study with Recommended Solutions †

**DOI:** 10.3390/healthcare13091006

**Published:** 2025-04-27

**Authors:** Sahra Ibrahimi, Sarah Yeo, Korede Yusuf, Zarah Akrami, Kevin Roy

**Affiliations:** 1 Department of Global Health, Denison University, 100 West College Street, Granville, OH 43023, USA; 2 University of Arizona Cancer Center, University of Arizona, Tucson, AZ 85719, USA; syeo@arizona.edu; 3 College of Nursing and Public Health, Adelphi University, Garden City, NY 11530, USA; kadegoke@adelphi.edu; 4 Milken Institute School of Public Health, George Washington University, Washington, DC 20052, USA; zakrami@gwmail.gwu.edu; 5 Department of Family Science, School of Public Health, University of Maryland in College Park, College Park, MD 20742, USA; kroy@umd.edu

**Keywords:** Afghanistan, maternal healthcare, perinatal health, primary data, the Taliban regime

## Abstract

**Background/Objectives**: This study identifies barriers to maternal and child healthcare access in Afghanistan under the Taliban and proposes solutions using the WHO Health System Building Blocks Framework. **Methods**: Midwives and mothers were recruited via chain-referral sampling. After obtaining IRB and the participants’ informed consent, in-depth virtual interviews, guided by Social Cognitive Theory, were recorded, transcribed, and analyzed using content analysis in MAXQDA 2020. **Results**: Data analysis revealed four primary consequences of the political unrest in Afghanistan that have exacerbated barriers to accessing maternal and child healthcare: (a) Taliban-imposed restrictions on women’s education and mobility, reducing female healthcare providers and limiting mothers’ access to care; (b) increased poverty, preventing women from attending perinatal visits due to out-of-pocket costs; (c) the deterioration of healthcare services, including medicine shortages, weakened health financing due to donor withdrawals, lack of insurance, and poor governance; and (d) the increased perpetuation of misinformation and harmful practices, such as the use of clergymen for medical advice instead of doctors. Using the WHO Health Systems Framework, we recommend solutions that address issues in service delivery, health workforce, health information systems, access to essential medicines, financing, and governance. **Conclusions**: This is the first qualitative study capturing Afghan mothers’ and healthcare providers’ experiences under Taliban rule. Our findings can inform international efforts to advocate for women’s healthcare and education rights and guide global aid programs in strengthening Afghanistan’s healthcare system in alignment with Sustainable Development Goal 5.

## 1. Introduction

The experience of more than four decades of conflict has impeded the development of the healthcare system in Afghanistan, and consequently, maternal and child health outcomes have worsened [1]. There were some improvements in access to healthcare between 2001 and 2021, mainly due to international donors and foreign aid [1]. For instance, the Basic Package of Health (BPHS), supported by donors and providing free-of-cost services for people, contributed to increased access, including maternal and child healthcare for the poor [2]. However, no health insurance or universal healthcare coverage (UHC) was available [1,2]. In 2019, approximately 79% of the total health expenditure came from households’ out-of-pocket spending, while the central government only contributed 8% through free services in public hospitals [3]. Although public hospitals offer free care, they lack advanced laboratories and pharmacies [1]. Often, patients are referred to private providers for lab tests and medication, which they must pay out of pocket [2]. Access to healthcare in rural areas is even worse [4]. In the past two decades, before the Taliban’s takeover in 2021, there has been considerable progress in community midwifery education, with the number of midwives in the country growing from 467 in 2003 to 20,000 in 2016 [5]. However, with the recent takeover of the country by the Taliban, numerous skilled providers and donors have left the country [6], the rate of unemployment and poverty has increased, and women’s education and mobility are restricted [7,8,9], making the health system even more vulnerable.

The low use of healthcare, particularly antenatal care (ANC) is a significant risk factor for an increased rate of adverse pregnancy outcomes [10,11,12] and maternal mortality [13]. Despite the global effort to reduce maternal and child mortality, a large proportion of Afghan women receive little to no antenatal care during pregnancy [4]. The 2015 Afghanistan Demographic and Health Survey (AfDHS), the first and only national survey of its kind, revealed that just 59% of Afghan women who gave birth in the five years preceding the survey received antenatal care (ANC) from a skilled provider [4]. The remaining 41% gave birth at home and did not access any antenatal care. Despite the World Health Organization (WHO) recommending a minimum of four ANC visits, only 18% of Afghan women attended four ANC visits in 2015 [4]. Since the Taliban’s takeover, the use of healthcare has worsened in the country [14].

Recent policies by the Taliban, including banning women’s higher education and mobility, have increased gender disparities [8,15]. These inequalities hinder female providers’ ability to train future providers and women’s ability to receive healthcare and/or have autonomy to make decisions about their health. In return, a lack of education and autonomy can potentially exacerbate domestic violence [16] and adverse maternal and child health outcomes [17]. Afghanistan is a low-income country with some of the highest rates of domestic violence [18] and maternal and child mortality [19,20]. In 2019, the neonatal mortality rate was 36 per 1000 live births, and for children under 5, the mortality rate was 60 per 1000 live births [19,20]. Recent policy changes have worsened these already challenging issues.

There are very limited studies assessing the status of maternal and child healthcare and healthcare utilization since the Taliban’s takeover in 2021. This study aims to identify factors hindering access to and the utilization of maternal and child healthcare in Afghanistan under the Taliban regime and to discuss recommended solutions based on data and the literature using the WHO Health System Building Blocks Framework. Beyond filling the current gap in the existing public health literature, our study will inform the international community’s efforts to pressure the Taliban into protecting women’s rights to healthcare and education, thereby reducing maternal and infant mortality in Afghanistan. This study will also provide insights for global aid programs aimed at improving healthcare infrastructure and accessibility, especially in rural areas, to save the lives of both mothers and their infants. At the national level, the findings can be translated into Dari and Pashto (Afghanistan’s two national languages) and shared with policymakers, as well as the public, enabling a better understanding of the significance of maternal healthcare and its impact on birth outcomes. Considering the current condition of women under the Taliban administration, this research holds significant relevance.

## 2. Methods

### 2.1. Approach and Theory

The health experiences of women in Afghanistan are complex, dynamic, and multidimensional. We used a qualitative approach, which allows for a comprehensive assessment and understanding of context, process, and meaning. Data were collected through in-depth virtual interviews using a semi-structured interview guide informed by Social Cognitive Theory [21]. Although the interview guide for the providers was different from the one for the patient, both guides consisted of questions about access to maternal healthcare, perceived barriers and facilitators, cultural practices around seeking care, the structure of the healthcare system (private vs. public, insurance vs. out of pocket, etc.), self-efficacy, and demographics. The interview guides were piloted by two Afghan volunteers, which further helped with the clarity, cultural relevancy, and appropriateness of the questions. A few additional questions were added based on new themes that emerged from the pilot. This study was approved by the Institutional Review Board of the University of Arizona (Protocol number: 2104716241) on 27 May 2021.

### 2.2. Recruitment and Data Collection

Five Afghan healthcare providers and seven patients were recruited using a chain-referral sampling method. We used chain-referral sampling because recruiting women under Taliban rule was extremely difficult. Many feared for their safety, even with confidentiality protections. This method allowed us to build trust, as participants were more likely to join after being referred by someone they knew. Data were collected through in-depth interviews (1–1.5 h) using a semi-structured interview guide from January 2023 to August 2023. The interviews were conducted through Zoom or WhatsApp calls. At the beginning of the interviews, the research study protocol was explained to the participants, and their informed verbal consent was obtained. Most participants were more comfortable speaking in Dari (their native language). Therefore, the first author, who was born in Afghanistan and is fluent in both Dari and English, conducted most interviews (10), and the second author conducted two interviews, one in English and one with the first author, serving as an interpreter. The interviews were audio-recorded and then transcribed in English. Finally, the transcripts were reviewed and double-checked for data accuracy.

We stopped at 12 participants for the following reasons: (1) The interviews were in-depth, and we gathered rich data from each participant. By the twelfth interview, we began to observe some data saturation, with participants sharing similar stories and little new information emerging. (2) It was difficult to find additional women willing to share their stories, as many were concerned for their safety. Virtual recruitment of women in rural areas was especially challenging due to limited internet access and inconsistent electricity.

### 2.3. Data Analysis and Data Quality

A list of potential codes was first developed based on the semi-structured interview guide informed by Social Cognitive Theory and its constructs. Additionally, the first and second authors read the transcripts to fully understand the data and discussed emerging codes based on the notes that each made. The newly emerged codes from the data were incorporated into the codebook, even if they were not originally part of the theory, to ensure a comprehensive understanding of the interview content. Based on the iterative process, the codebook was finalized, and the first author coded all the remaining transcripts based on the agreed codebook.

The coding process resulted in 60 codes, which we categorized under the following four major emerging themes that influence access to healthcare: discrimination against women under the Taliban regime; increased poverty; worsening of the health system; and a lack of health literacy and increased perpetuation of misinformation. Finally, we used the WHO Health Systems Framework [22] to recommend solutions that address issues in service delivery, health workforce, health information systems, access to essential medicines, financing, and governance. The triangulation of patients and providers as multiple sources of data and the utilization of multiple coders are selective techniques that enhance the rigor and trustworthiness of our data [23].

## 3. Results

Table 1 describes the participants’ characteristics (n = 12). Most participants lived in Afghanistan (n = 11) except for one recently arrived Afghan refugee who resided in the U.S. Five of the twelve participants were providers, and the remaining seven were patients. Most providers were Hazara (60%), lived in Kabul (80%), worked full-time (80%), and had four or more years of experience in the medical field. Most of the providers had a bachelor’s degree in midwifery (80%). Only one had a medical degree and served as a chief gynecologist. All patients lived in Afghanistan and belonged to either Tajik, Pashtun, or Hazara tribes. About 71% of the patients had three or more children and were housewives, while the remaining worked part-time. One of the patients was uneducated, three had some high school education, and the rest had a bachelor’s degree.

**Figure 1:**
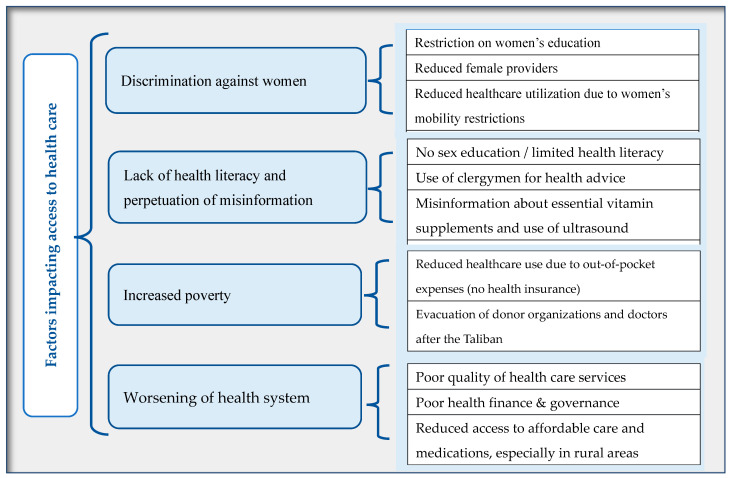
The emerging themes and factors hindering access to healthcare in Afghanistan.

Figure 1 illustrates the emerging themes and factors hindering access to maternal healthcare in Afghanistan. Each theme and the relevant data are described in the following four sections.

### 3.1. Discrimination Against Women

The Taliban has restricted women’s fundamental rights, including receiving an education and traveling unless women are escorted by a male relative. These restrictions on women’s mobility in some regions of the country have hindered patients’ access to healthcare. A patient shared that her friend could not see a doctor because her husband was not home to escort her to the clinic.

My friend who lives in Kandahar Providence has got pregnant, but she cannot go to a clinic by herself because, in her village, the Taliban punishes women who are outside without their husbands.

Another patient shared that women’s access to healthcare became limited because they could not afford to pay the medical fees as they were banned by the Taliban from working and having professional jobs.

Some women had jobs and could afford to visit a doctor, you know. Now, women are banned from work and from having a job, and even some men also struggle with finding a job because of the worsening of the economy.

In addition, female health providers shed light on other gender-based discrimination they endured in their professional lives. For example, they endured daily attire-related harassment and safety concerns under the current role of the Taliban. More significantly, this discrimination led to restrictions that have limited the education and training of future midwives. A physician and chief gynecologist who had 12 years of clinical experience stated the following:

I conducted major surgeries and trained new physicians and midwives; however, now I can no longer teach or train because women are banned from educating or receiving an education, but I still practice as a gynecologist.

Midwives also shared their concerns about the lack of advanced training opportunities offered in foreign countries. Due to the political changes, traveling outside the country has become challenging. One provider recounted the following:

As a healthcare professional, I am eager to learn new medical procedures and strengthen my skills. Before the Taliban, I used to have the opportunity to travel to other countries like India and Nepal [for training], but now, with the Taliban in power, I do not get those opportunities.

As a result of these restrictions and frustrations, many female providers have been prohibited from providing services, or perhaps have been forced to curtail their professional services. This is extremely problematic in communities where religious and conservative men do not allow their wives to see a male doctor.

Religion plays a big role. A male doctor cannot touch a female patient. Several patients, especially their husbands do not agree with a male doctor seeing their wives. In a society where women’s education is banned, and future doctors cannot be trained, how could there not be a lack of female healthcare providers, and how could mothers not die?

Ultimately, midwives shared their concerns about the increased rates of mortality due to a lack of female doctors, as many educated health professionals left the country after the Taliban took control. Those who have remained in the country do not feel safe going to work because women are banned from working outside. A midwifery trainer, who was also the Director of the Provincial Midwife Association, shared the results of her survey conducted in partnership with Jhpiego. She reported that their findings showed an increase in the maternal mortality rate due to the lack of access to healthcare, as well as the harassment of midwives by the Taliban.

…These reports show how much maternal health was affected by the Taliban. They, of course, caused an increase in maternal mortality rate because of the lack of access to health services… 81% of midwives claimed that they become harassed by the Taliban [for going to their jobs].

### 3.2. Lack of Health Literacy and Perpetuation of Misinformation

Before the Taliban regime gained power, community health workers had established a set of best practices to provide outreach and enhance the literacy of women across the country. This accomplishment was dramatic and included workshops in hospitals and mosques to educate women. One provider recalled the following:

At least before the Taliban, we could provide free workshops to educate pregnant women…we used to teach women about hygiene, breast cancer, and iron supplements. We used to provide these not only in our hospitals but also in mosques. But now none of those happen or can happen because of the Taliban.

Restrictions on women’s engagement with female providers and the discouragement of this nascent system of outreach have led to a retrenchment of inaccurate health beliefs that can prove harmful to women throughout the country. For example, one woman reflected on her personal experiences with menstruation and the lack of support within her family.

I also hope that there will be more awareness and education about women’s health. Many girls do not know that getting their menstruation cycle is normal. When I first got mine, I cried, and I was scared. There is no education about it at school. I was shy to tell my mother about it, so I told my sister, and she said that it was okay and normal.

In particular, widespread misinformation about how to handle pregnancy disregards advances in technology and science and prevents women from obtaining the proper treatment. For example, a provider argued that some women believe that “ultrasound is bad for the fetus and that it prevents the fetus from growing. Some others think that it [ultrasound] causes loss of hair in a fetus. Some other women tell us that their mother-in-law has forbidden them from ultrasound”. Others indicated that critical vitamin supplements were regularly denigrated by women in local communities.

Perhaps the most damaging were superstitions about common developments during pregnancy. A midwife mentioned that “some pregnant women are severely anemic and thus experience white patches on their face skin, but because they are uneducated, they believe it is normal and do not know that it is due to low iron”. She also pointed out that others believed that antibiotics dry out fetuses, and so women are discouraged from taking them. The consequences of unproven home-grown remedies can be harmful to women and fetuses. Another provider recounted a dangerous episode:

Once, I had a patient with high blood pressure who left the hospital right after birth, and when she got home, the family members fed her butter and salty food. She had several heart attacks, and they took her to a Mullah for the day. By nighttime, she went unconscious, and they had to bring her back to the hospital… These types of incidents happen often in Afghanistan.

Other superstitions have served to distract women from focusing on their own experiences of healthy pregnancy and to shift power to traditional beliefs that are embedded within intergenerational family relationships. Another midwife stated, “Some mothers-in-law paint the feet of pregnant women with the blood of chicken because they believe that this will help with a smooth and fast birth. Some others use a plant, *Panja*, as a predictor of good birth”.

The Taliban has not only hindered health literacy through media or educational workshops that impact access to maternal healthcare but has also caused an increase in the perpetuation of misinformation through religious extremists in mosques. In addition to their families, women are left to contend with the authority of local religious leaders, who are men and who can determine the course of treatment for pregnancy. A provider noted the misdirection of saying that “if they [women] do not go to a Mullah, their pregnancy might be cursed by other women”. Another provider pointed out that

…Mullahs advise women to visit mosques and see them instead of seeing a doctor. Some of the Mullahs threaten women that if they go to a doctor, something bad may happen to them, and so women get discouraged from going to a doctor, especially if they are uneducated.

An influential gynecologist argued that, as a result of such misinformation, “several women die of pregnancy because of their lack of awareness about the importance of perinatal healthcare visits, the process of pregnancy, family planning, and hygiene”. This practitioner further highlighted the lack of awareness and health literacy among women, especially in rural areas, and how the Taliban’s administration has become a barrier to educating the population about women’s health issues, including family planning. Before the Taliban, Afghanistan’s health sector had suggested that a pregnant woman should visit a doctor every four weeks until week 32 and then every two weeks from that point on.

We used to publicize this through the media to bring awareness. We also broadcasted health education clips, and this was especially helpful in reaching people in rural areas, but now, with the Taliban, we cannot do any of these things. Women with hypertension need to meet a doctor frequently during pregnancy so that she doesn’t end up with preeclampsia, but unfortunately, now doctor visits are very less likely.

Perhaps the only women who still receive access to quality healthcare are educated women with families who have resources. These women were more likely to access healthcare than uneducated women. As one patient indicated,

My husband, who is a doctor, and I knew the importance of perinatal care; I would get checkups regularly including ultrasound… but I know several women who did not go to a doctor unless they got severely sick.

### 3.3. Increased Poverty

Under the Taliban’s rule in Afghanistan, the worsening of the economy and the lack of health insurance have restricted people’s ability to obtain essential healthcare services and medications, as they are required to pay medical bills out of pocket. Several participants expressed that “there is no health insurance, and people pay out of pocket, which is why, with the worsening of the economy, people don’t use health services unless they are severely sick or nearing death”.

The stark differences in the impact of poverty on women’s health were clear in the constant comparisons that both women and providers made in their reflections. A clear consequence of having new financial constraints in a restricted healthcare system is the delay in seeking treatment—or even securing treatment at home. A provider was very concerned about this rational but dangerous option.

Sometimes they [patients] wait to make enough money, let’s say, like 50 Af., and then, they come to the doctor, which is too late, and their health is much worse. Sometimes they do come to doctor, and sometimes they even don’t come to doctor…they just go directly to a pharmacy, and they get the medication from a pharmacist because they do not have money for both medication and doctor.

A patient echoed almost the same themes but also disclosed the turn to unproven home remedies for health concerns.

Before the Taliban, people could afford healthcare, but now the economy is so bad that people do not visit healthcare and instead try to cure themselves at home with home remedies. The cost of medications and lab tests has also risen.

Related to the limited decisions about healthcare that economically marginalized patients now make under the Taliban, the choice about the utilization of hospitals was critically different. Before the Taliban’s rule, private hospitals were preferred over public hospitals due to less crowding and better services. However, the economic downturn from that time has forced most people to lose access to care or wait for hours in line in overcrowded public hospitals. A patient asserted that “with the Taliban take over, people cannot afford going to public hospitals, let alone to a private one”.

Similarly, a provider described how her hospital has adapted to continue providing services despite Taliban restrictions and poverty.

Before the Taliban, people preferred and were able to use private hospitals because private hospitals had better quality of care and services than public hospitals. Public hospitals are also very crowded because they are cheaper; for example, the fee of a visit is 20 Af. vs. in private hospitals, which can be up to 200 Af. … In our hospital, we provide free services two days a week because otherwise, there won’t be enough patients visiting. We also provide a 30% discount. People are more likely to use services when the costs are lower.

### 3.4. Worsening of the Health System

Patients and providers both strongly agreed that under Taliban rule, the health system in Afghanistan has worsened in terms of service delivery, health workforce, the provision of medications, and health financing. The participants highlighted the following challenges in public and private hospitals.

In public hospitals, women are discharged quickly because of high demand and crowdedness, and in private hospitals, women themselves leave sooner because the longer they stay, the more it will cost [them]. In public hospitals, women have to leave within an hour of delivery. In private hospitals, they usually stay up to 4 h or so.

Despite longstanding differences in access and quality of care, the conditions of public hospitals have suffered dramatically in recent years. Patients experience poor and inadequate service delivery due to high demand for healthcare, leading to almost no access to prenatal checkups. One provider shared an anecdote about how the process for checkups occurs in public hospitals:

There are no prenatal checkups because their capacity is so small that if a woman goes there and says, “I need a checkup”, they would laugh at her. They would be like, “We have women here dying because they need care. And you just come here for a checkup, like, you look fine, just go home unless you have a severe problem and it’s like your baby’s coming out”…Also, the facilities are not good. It’s cold, and there are not enough beds.

Additionally, two women discussed their challenges, which they believed to be very common across the country. One woman insisted that “if you go to private clinics or hospitals, they try to charge you for unnecessary lab tests. They try to make more money. On the other hand, public hospitals are free, but they treat people very badly”. Her friend expanded on this concern by discussing the inadequate appointment system.

In public hospitals, there is no system for making appointments. People just show up and stay in line for health services. I saw a woman who kept saying that “the baby is coming, the baby is coming!”, but because she was in the waiting line, there was no bed for her, and the doctors were not paying attention. She gave birth in the hallway, on the cold concrete. There are not enough doctors, so doctors get tired, and when it is their lunchtime, they go to lunch and don’t care if patients are in pain and need the doctor. Some doctors yell at patients, saying if you couldn’t bear the pain, you shouldn’t have gotten pregnant.

Furthermore, the providers indicated how the healthcare system in rural areas has significantly deteriorated in comparison to health services in cities like Kabul. In rural areas, the scarcity of providers and a lack of incentives for providers to deliver services in those regions has permanently harmed women’s access. The Taliban government provides no incentives for female midwives and also discourages them from practicing midwifery. In Kabul City, midwives are required to be licensed, but in the rural areas, some people practice as doctors or midwives with no education or licenses. A provider stated the following:

Good doctors don’t like to live in rural areas because of poor living conditions, lack of technology, and security issues. Before the Taliban, there were some incentives provided to doctors to work in rural areas. For example, a midwife was paid 7000 a month in Kabul, and in rural areas, they were paid 30,000. Now that the Taliban are here, things have gotten worse. There is no incentive, and women face many obstacles in practicing midwifery.

Medication shortages have also gripped the current healthcare system, especially in the public health sector and rural areas. Public hospitals have also run out of medications quickly because they have more patients than private hospitals. In rural regions, distance and weather conditions hinder access to medication, leaving patients stranded and without essential medications. A provider noted that “In rural areas, when hospitals run out of medicine in the winter… They cannot travel to the city to get them because the snow blocks the road for 6 months or more”.

Overall, the system has been impacted by the withdrawal of support from non-profit and donor organizations. These groups evacuated with the reemergence of the Taliban regime. A midwife made an obvious observation that “educated health professionals, as well as experienced and skilled doctors, left Afghanistan after the Taliban took over”. A healthcare provider highlighted the past presence of non-profit organizations in Afghanistan.

Before the Taliban, there were some non-profit foreign organizations that were financially supporting the healthcare system; although, because of governmental corruption, not all the benefits reached the beneficiaries. But it was much better than the current situation under the Taliban.

## 4. Discussion and Recommendations

### 4.1. Summary of Findings

Discrimination against women and the violation of women’s rights under the current Taliban regime have prevented women from receiving an education, hindering both the training and enhancement of current providers’ skills and the establishment of the next generation of female providers. The restrictions on women’s mobility and women’s fear of harassment by the Taliban have made it difficult for female providers to deliver health services and for patients to access those services. Furthermore, the Taliban has banned health literacy initiatives that previously took place through media and educational workshops. For instance, before the Taliban’s control, the importance of perinatal care visits was publicized through mass media and educational workshops; however, those activities are now banned, and women are encouraged to seek medical advice from Mullahs and clergymen with no health background or training. This lack of health literacy has intensified misinformation, such as women believing that ultrasounds and vitamin supplements are dangerous for their babies and that without consulting a Mullah, their pregnancy will be cursed.

Additionally, providers and patients themselves argue that due to poverty, decreased donor funding, and increased out-of-pocket medical expenses, women are reluctant to seek healthcare unless they are severely ill or nearing death. The cost of medications and lab tests has also risen significantly. As a result, some patients reported delaying seeking healthcare, while others reported attempting treatments at home. Furthermore, since the Taliban takeover, Afghanistan’s health system, including health governance and financing, health service delivery, the health workforce, and the provision of medications, has declined and worsened in quality. All of these factors have had an adverse effect on patients’ healthcare accessibility and their overall healthcare experience. Most of the patients reported negative experiences in both private and public hospitals; for example, overcharging for unnecessary tests in private hospitals, poor service delivery, insufficient facilities, and disrespectful treatment in public hospitals. In rural areas, healthcare access is worse due to Taliban policies that discourage female midwives and provide no incentives for skilled providers to serve in rural areas. The healthcare system has been further weakened by the departure of numerous educated healthcare professionals and donor organizations.

### 4.2. Recommended Solutions

**Figure 2:**
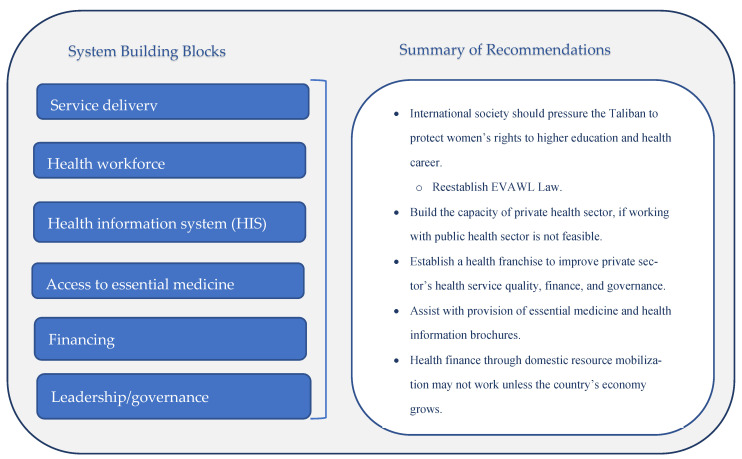
The system building blocks of the WHO Health System Framework and our recommendations for strengthening those building blocks in Afghanistan.

The WHO framework delineates six fundamental elements in health systems, referred to as building blocks. These elements are service delivery, health workforce, health information systems, access to essential medicines, financing, and leadership/governance [22]—all of which impact access to healthcare and health outcomes. Using this framework, we can make recommendations about Afghanistan’s maternal healthcare based on the observed data and the literature. Figure 2 presents the system building blocks of the WHO Health System Framework and provides a summary of our recommendations for strengthening those building blocks in Afghanistan to enhance access to maternal healthcare.

Service delivery quality and the midwifery health workforce depend on women’s education, training, and mobility. Because Afghanistan is an Islamic country, and many women are uncomfortable or not allowed to see a male health provider [24], female health providers are a crucial part of the health workforce. The number of female health providers is already declining; this has also impacted the quality of service [25]. As the participants mentioned, public gynecology clinics and hospitals are crowded with too many patients and insufficient health providers. The international community needs to pressure the Taliban to protect women’s rights, especially their rights to education and professional jobs.

Protection of women’s right to healthcare will also alleviate the problem of poverty and make healthcare affordable. As the participants shared, before the Taliban, both men and women could contribute to their families, but now, the entire family depends on one person’s income, forcing them to prioritize food and shelter over health checkups or other preventative healthcare. Studies show that women’s lack of engagement in the economy and productivity also impacts a nation’s overall economic growth and health spending [26]. The Elimination of Violence Against Women Law (EVAWL), which was established in 2009 by the United Nations (UN) but dismantled in 2021 by the Taliban, needs to be reestablished and enforced to protect women and their well-being [27]. In the meantime, if health awareness and literacy are not possible through mass media, health brochures should be distributed in communities to fight against misinformation.

Before the Taliban, several donors worked with Afghanistan’s Ministry of Public Health (MoPH) to strengthen the public health sector’s capacity for health system information, provision of medications, and health finance and governance [28]. However, now, several donors have left Afghanistan, and many foreign countries do not recognize the Taliban as a legitimate government [29]. The other issue is that many educated MoPH personnel left the county after the Taliban’s takeover, and most of those personnel were replaced by the Taliban [30], many of whom have no education. The donor organizations that still want to help Afghanistan’s health sector but do not want to work with the Taliban should focus on working with the private health sector and strengthening their capacity, as this has been an effective method in other developing countries [31].

One recommendation to improve and standardize the private sector’s health service quality, finance, and governance is to establish a health franchise network. A study in Nepal showed that the involvement of private providers within a franchise network contributed to improved clients’ perception of the quality and accessibility of health services [32]. Another study found a positive association between franchising reproductive health services and increased patient satisfaction, increasing providers’ dedication to delivering quality service in the underdeveloped rural regions of a developing nation [33]. Furthermore, donor organizations and foreign aid should also focus on the provision of medicine, especially family planning methods, because the Taliban is against family planning and the use of contraception [34]. This will be useful, as research has shown that those who experience spousal violence are more likely to use family planning [35]. Additionally, community-based distribution of contraceptives has been shown to improve access to family planning methods [36].

These family planning products and medicines need direct funding from donors since Afghanistan’s economy does not have the capacity to mobilize its domestic resources for health spending. Although domestic resource mobilization (DRM) and financing health through taxes have worked in some countries to finance health [35], DRM may not work in Afghanistan in the short term due to corruption and its poor and informal economy. An informal economy refers to the segment of an economy that operates without government taxation or monitoring [37]. Studies show that without strengthening governance and financial management, DRM as a mechanism for financing health in low-resource countries has been unsuccessful [38].

## 5. Conclusions

Afghan women are enduring several barriers to accessing maternal and child healthcare in Afghanistan due to their restricted mobility and access to education, the perpetuation of misinformation, increased poverty, and the worsening of the health system. To improve access to maternal healthcare, the international community and donor organizations should focus on protecting women’s rights to education and employment, developing the capacity of the private health sector through building a franchise, and assisting with providing medicine and other health commodities.

## Figures and Tables

**Table 1:** Characteristics of study participants; n = 12 (midwifes = 4, physician = 1, and patients = 7).

Characteristics		Providers n (%)	Patients n (%)
Age (mean, range)	35 (25–53)		
Ethnicity	Tajik	1 (20)	3 (43)
	Pashtun	1 (20)	2 (29)
	Hazara	3 (60)	2 (29)
Education level	No education	0 (0)	1 (14)
	Some high school	0 (0)	3 (43)
	Bachelor’s degree	4 (80)	3 (43)
	Medical degree	1 (20)	0 (0)
Employment status (4+ years of work experience)	Full-time	4 (80)	0 (0)
	Part-time	1 (20)	2 (29)
	Housewife	0 (0)	5 (71)
Current place of residence	Afghanistan	4 (60)	7 (100)
	Recent refugee (U.S.)	1 (20)	0 (0)
Number of children	None	2 (40)	0 (0)
	Two	2 (40)	2 (29)
	Three or more	1 (20)	5 (71)

## Data Availability

This study used primary qualitative data. The data may be shared privately upon reasonable request.
